# Editorial: Targeted Subcellular Delivery of Anti-cancer Agents

**DOI:** 10.3389/fphar.2018.01577

**Published:** 2019-01-22

**Authors:** David A. Jans, Alexander S. Sobolev

**Affiliations:** ^1^Department of Biochemistry and Molecular Biology, Nuclear Signalling Laboratory, Monash Biomedicine Discovery Institute, Monash University, Clayton, VIC, Australia; ^2^Laboratory of Molecular Genetics of Intracellular Transport, Institute of Gene Biology, Russian Academy of Sciences, Moscow, Russia; ^3^Faculty of Biology, M. V. Lomonosov Moscow State University, Moscow, Russia

**Keywords:** drug delivery, compartmentalization, organelles, nuclear targeting, mitochondrial targeting

## Strategies For Targeted Cancer Therapy

One of the leading causes of death worldwide, cancer stems from accumulated and/or inherited genetic abnormalities, leading to prolonged cell survival, resistance to normal cellular death and an increased propensity for the cell to proliferate in uncontrolled fashion. Understanding of the molecular mechanisms underpinning many cancers has progressed, but treatment remains largely focused on eradicating tumor cells from the patient using a therapeutic agent(s)/procedure which almost always causes the unwanted death of surrounding normal bystander cells (Wagstaff and Jans, 2009; Stelma et al., 2016). Further, through the nature of the majority of the drugs/strategies used, it is the highly proliferating normal cells of the body that tend to be most susceptible to this effect, including cells within the bone marrow and the immune system, with the end result being a severely immunocompromised patient who is highly susceptible to infection by life-threatening pathogens (Stelma et al., 2016).

Since tumor cells derive from normal cells of the body, selectively killing tumor but not normal cells by exploiting a tumor cell's “unique” properties can be challenging. Tumor cells may be particularly sensitive to a toxic agent (e.g., radioactive iodine in the case of differentiated thyroid cancer–Luster et al., 2008), or “addicted” to elevated levels of a cellular activity such as that of protein kinase CK2 (Franchin et al., 2017) or the nuclear transport molecule importin β1 (Kuusisto and Jans, 2015), and hence more sensitive than normal cells to inhibitors of this activity. Finally, tumor cells may express/overexpress particular cell surface components (e.g., EGF receptor), enabling highly efficient delivery of highly toxic agents/treatments to tumor and not normal cells through receptor-mediated delivery (e.g., Rosenkranz et al.). Combining multiple tumor-specific/-enhanced moieties into a single modular multifaceted approach, such as modular nanotransporters (MNTs)/modular nanocarriers, can enable the desired anticancer outcomes, without unwanted effects on normal bystander cells (Glover et al., 2009; Wagstaff and Jans, 2009; Sobolev).

A variant on the theme of exploiting key tumor cell sensitivities is to deliver toxic agents to particular hypersensitive subcellular compartments of the cell, such as the nucleus or mitochondria. Clearly, DNA encoding a toxic gene product to be expressed in the target tumor cell, or agents acting on the DNA of the tumor cell need to be delivered to the nucleus for toxic effects to be exerted (Durymanov and Reineke); similar considerations apply to agents specifically targeted at limiting mitochondrial respiratory or redox function (Battogtokh et al.). Antitumor agents generally capable of cytotoxic effects in any part of the cell may have particular “subcellular tropisms” for hypersensitivity, enabling lower doses of a drug to effect cell killing; examples are photosensitizers, radionuclides emitting short-range particles such as alpha-particle- or Auger-electron emitters (Bavelaar et al.; Rosenkranz et al.; Sobolev).

In this Research Topic, leading experts in the area of drug delivery present their most recent findings and summarize new ideas to show the way forward in developing strategies to deliver drugs efficiently to specific subcellular sites, with the aim to kill cancer cells selectively. The articles summarize in various ways the benefit of delivering certain groups of anti-cancer agents/treatments into specific subcellular compartments, and how to achieve this with respect to the nucleus, as well as different cytoplasmic compartments, including the mitochondrion. The antitumor agents discussed include theranostic radionuclides, together with state-of-the-art techniques for detecting them *in situ* (Bavelaar et al.), nucleic acids (Durymanov and Reineke), Auger electron emitters (Rosenkranz et al.; Sobolev), therapeutic antibodies (Slastnikova et al.), mitochondrial-targeting drugs (Battogtokh et al.), and the mTORC1 complex targeting agent rapamycin (Peddi et al.). Delivery vehicles showcased include radiolabeled pharmacons (Bavelaar et al.), nanoparticles with genetic payload (Durymanov and Reineke), MNTs (Rosenkranz et al.; Sobolev; Glover et al., 2009), protein-transduction domains, or liposome/vesicles (Slastnikova et al.), mitochondrial targeting nanocarriers (Battogtokh et al.), and FKBP-elastin-like peptide conjugates that are taken up by macropinocytosis (Peddi et al.).

## Nanocarrier-Mediated Drug Delivery Into Cellular Subcompartments

Drug delivery to specific subcellular compartments requires passage through a number of barriers that are impermeable to most types of molecules. The first barrier is the lipid bilayer of the cell membrane; whilst physical methods such as electroporation and gold particle bombardment can be used, directed entry can occur through protein transduction conferred by cell penetrating peptide moieties (see Wagstaff and Jans, 2006; Slastnikova et al.) leading to direct access to the cytoplasm. Receptor-mediated endocytotic delivery can exploit the receptor repertoire of the tumor cell surface, although exit from the endosome is then a necessary step for cytoplasmic access, usually requiring an endosomal escape mechanism (Durymanov and Reineke), e.g., through components such as diphtheria toxin (Rosenkranz et al.; Sobolev). Agents delivered using liposomes/vesicles/virus-like carriers (Slastnikova et al.) or macropinocytosis (Peddi et al.) usually accumulate in intracellular vesicles/endosomes, again, similarly requiring escape from the intracellular vesicle to access the cytoplasm. Once the cytoplasm has been accessed, specific targeting signals for delivery to the nucleus (see Pouton et al., 2007; Durymanov and Reineke), mitochondrion (see Battogtokh et al.), or other organelles confer ultimate delivery to the appropriate subcellular compartment that is hypersensitive for toxic action.

## Modular Nanocarriers

MNTs/modular nanocarriers (Rosenkranz et al.; Sobolev; Glover et al., 2009) include targeting modules/drug or DNA binding domains/tracking labels to be able to mediate all of the steps of cell entry, endosomal exit (if required), and subsequent monitored targeting to the target organelle. MNT systems (Slastnikova et al., 2012; Sobolev) have a number of important advantages. Since they can achieve high local concentration of therapeutic agents at a hypersensitive cellular site, the therapeutic dose administered can be decreased to reduce unwanted bystander effects/toxicity/immunogenicity; MNTs have other advantages such as ease of production, uniformity of physico-chemical properties, and nanoparticle size that does not preclude translocation through nuclear pores etc. Importantly, because of the modular design, the MNT platform is an exciting prospect for personalized therapeutics, where ligand and drugs delivered can be tailored to the individual patient.

Theranostic agents combine treatment and diagnostics, importantly enabling “real time” monitoring of therapeutic efficacy, as well as tumor progression (Bavelaar et al.). Targeting theranostic agents to specific subcellular sites within a tumor cell affords many new avenues for the future, especially in an MNT design.

Drug delivery using modular nanocarriers, and exploiting cellular mechanisms for subcellular targeting is an important, developing area in the anti-cancer arsenal. The articles in this Research Topic give key insight into the many possibilities currently employed to achieve targeted subcellular delivery in a cancer context, and provide a strategic platform for endeavor in this vibrant research area in the future.

## Author Contributions

All authors listed have made a substantial, direct and intellectual contribution to the work, and approved it for publication.

### Conflict of Interest Statement

The authors declare that the research was conducted in the absence of any commercial or financial relationships that could be construed as a potential conflict of interest.
